# Dental Resin-Based Luting Materials—Review

**DOI:** 10.3390/polym15204156

**Published:** 2023-10-19

**Authors:** Aleksandra Maletin, Milica Jeremić Knežević, Daniela Đurović Koprivica, Tanja Veljović, Tatjana Puškar, Bojana Milekić, Ivan Ristić

**Affiliations:** 1 Faculty of Medicine, University of Novi Sad, 21000 Novi Sad, Serbia; milica.jeremic-knezevic@mf.uns.ac.rs (M.J.K.); daniela.djurovic-koprivica@mf.uns.ac.rs (D.Đ.K.); tanja.veljovic@mf.uns.ac.rs (T.V.); tatjana.puskar@mf.uns.ac.rs (T.P.); bojana.milekic@mf.uns.ac.rs (B.M.); 2 Faculty of Technology, University of Novi Sad, 21000 Novi Sad, Serbia; ivan.ristic@uns.ac.rs

**Keywords:** luting material, dental composite, resin-based composite, polymerization

## Abstract

As cementation represents the last stage of the work involved in making various indirect restorations (metal ceramic crowns and bridges, full ceramic crowns and bridges, inlays, onlays, and fiber posts), its quality significantly contributes to the clinical success of the therapy performed. In the last two decades, the demand for ceramic indirect restorations in everyday dental practice has considerably increased primarily due to the growing significance of esthetics among patients, but also as a result of hypersensitivity reactions to dental alloys in some individuals. In this context, it is essential to ensure a permanent and reliable adhesive bond between the indirect restoration and the tooth structure, as this is the key to the success of aesthetic restorations. Resin-based luting materials benefit from excellent optical (aesthetic) and mechanical properties, as well as from providing a strong and durable adhesive bond between the restoration and the tooth. For this reason, resin cements are a reliable choice of material for cementing polycrystalline ceramic restorations. The current dental material market offers a wide range of resin cement with diverse and continually advancing properties. In response, we wish to note that the interest in the properties of resin-based cements among clinicians has existed for many years. Yet, despite extensive research on the subject and the resulting continued improvements in the quality of these materials, there is still no ideal resin-based cement on the market. The manuscript authors were guided by this fact when writing the article content, as the aim was to provide a concise overview of the composition, properties, and current trends, as well as some future guidelines for research in this field that would be beneficial for dental practitioners as well as the scientific community. It is extremely important to provide reliable and succinct information and guidelines for resin luting materials for dental dental practitioners.

## 1. Introduction

**Figure 1:**
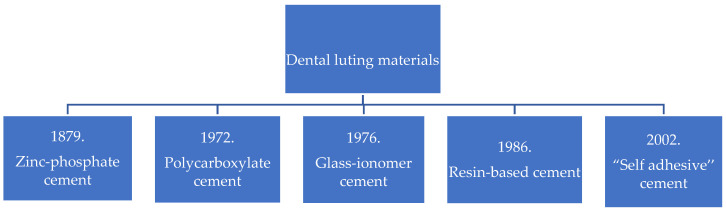
Development of dental luting materials throughout history (based on the data provided in articles cited under the reference numbers [3,4]).

For decades, zinc-phosphate cement from the group of conventional cements had been the clinical practitioners’ material of first choice for cementing restorations comprising gold and non-precious alloys. This approach has been largely successful, as confirmed by the results of some clinical trials suggesting restoration longevity of up to twenty years [1], motivating its use for cementing crowns and bridges [2]. However, in the early 1970s, polycarboxylate and glass-ionomer cements were introduced into the market, and in 2002 Hecht and Ludstech developed a new group of adhesive cements based on composite resins (resin cements) for use in ceramic restorations (Figure 1).

Studies have demonstrated their good mechanical properties, reliable adhesive bond with the tooth structure and restoration, easy handling and satisfactory aesthetics [3,4,5]. Adhesive cements are considered an improved version of luting materials due to their superior adhesion to the tooth structure [2]. As cementation represents the last stage of the work involved in fabricating indirect restorations (metal ceramic crowns and bridges, full ceramic crowns and bridges, inlays, onlays, and fiber posts), the quality of this procedure significantly contributes to the clinical success of the therapy performed [6]. Thus, due to the insufficient understanding of the chemical, physical and biological properties of luting materials commonly used in dental practice, this seemingly simple procedure can compromise the outcome of the performed dental procedure, thereby reducing restoration longevity [7]. The clinical success of restorative procedures is established, among other factors, by the degree of microleakage between the indirect restoration and the tooth structure several years after cementing the restoration. As microleakage increases the risk of secondary caries, postoperative sensitivity, compromised pulp integrity and tooth vitality, as well as dental plaque accumulation, every effort should be invested in mitigating this undesirable phenomenon [8].

Self-adhesive resin cements include new methacrylate monomers with phosphoric acid groups with the aim of enabling a self-adhesive reaction between this cement type and the tooth structure. Owing to their application, a low pH value and hydrophilic properties can be attained at the onset of the setting process. In the subsequent stages, the negatively charged monomer groups bind to the Ca^2+^ ions within the tooth, which-in combination with the alkaline part of the filler-facilitates a neutralization reaction [9]. This is one of the goals of the extensive research in the field of dental luting materials, which has resulted in improved luting agents, especially with the development of modern resin adhesive cement [10].

Resin luting agents have the capacity to achieve an adhesive bond with predominantly glass ceramics, low-filled glass ceramics and some of the intermediate-filled glass ceramics and intra-canal fiber posts [11]. As the adhesive bond between the ceramic restoration and the tooth structure is considered a key factor in the success of any restorative procedure, these findings are highly beneficial [10,12]. Extant studies further show that the application of dental luting materials based on composite resins also improves the fracture strength of the ceramic material, due to the positive influence of their mechanical properties (flexural strength, hardness) on the fracture strength of the restored tooth and the resistance to cracking of the ceramic restoration (Table 1) [3,13].

The interest in the properties of resin-based cement among clinicians has existed for many years. Yet, despite extensive research on the subject and the resulting continued improvements in the quality of these materials, there is still no ideal resin-based cement on the market. The manuscript authors were guided by this fact when writing the article content, as the aim was to provide a concise overview of the composition, properties, and current trends, as well as some future guidelines for research in this field that would be beneficial for dental practitioners as well as the scientific community.

## 2. Resin-Based Dental Luting Materials

In the last two decades, the demand for ceramic indirect restorations in everyday dental practice has considerably increased [8] primarily due to the growing significance of esthetics among patients, but also as a result of hypersensitivity reactions to dental alloys in some individuals [14]. In this context, it is essential to ensure a permanent and reliable adhesive bond between the indirect restoration and the tooth structure, as this is the key to the success of aesthetic restorations [15]. According to Fleming, the synergistic bond between the adhesive resin-based cement with the tooth’s support structure and ceramic restoration work together in a way that strengthens and enhances the performance of the entire system, resulting in an effective outcome for the overall dental restoration [16]. Yet, at present, there is no consensus on the most appropriate cementation protocol for ceramic restorations [17]. Namely, empirical evidence from dental practice shows that both conventional cement and resin-based can be successfully used for cementing polycrystalline ceramic restorations [18]. Still, some practitioners favor conventional cements as their application does not require any pretreatment of dental tissues or the use of specific handling protocols. These cements also exhibit high tolerance to the presence of moisture. However, the bond between the restoration and the tooth structure weakens over time. On the other hand, resin cements benefit from excellent optical (aesthetic) and mechanical properties, as well as from providing a strong and durable adhesive bond between the restoration and the tooth. For this reason, resin cements are a reliable choice of material for cementing polycrystalline ceramic restorations [17,19].

### 2.1. Resin-Based Dental Luting Materials—Chemical Structure

#### 2.1.1. Organic Matrix

**Figure 2:**
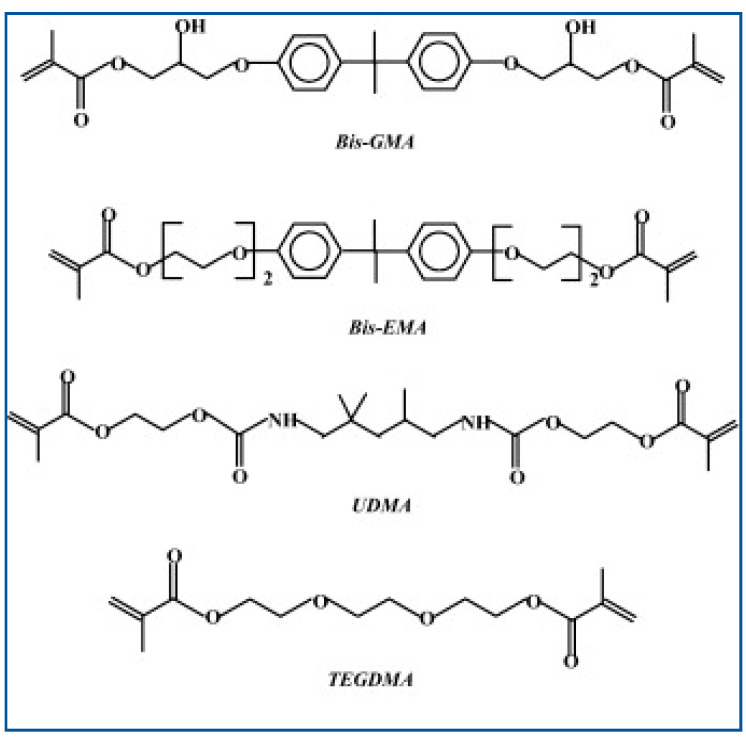
Chemical structure of resin-based luting material-type of the organic matrix [22].

The structure of resin-based cement consists of an organic resinous matrix, inorganic filler particles and silane [20]. The organic resin matrix primarily comprises dimethacrylate monomers such as bisphenol-A-glycidyl methacrylate (BisGMA), bisphenol-A-ethoxy dimethacrylate (BisEMA) and/or urethane dimethacrylate (UDMA) (Figure 2) [21,22], which contributes to the outstanding mechanical properties, fast polymerization reaction and low degree of polymerization contraction in resin cement [23]. In order to reduce viscosity and increase filler content, resin cement may also contain low-molecular monomers, so-called diluents, such as triethylene glycol dimethacrylate (TEGDMA) and ethylene glycol dimethacrylate (EGDMA) [21]. However, in BisGMA, the addition of diluents reduces the viscosity but also increases monomer crosslinking, which is not desirable, since it may promote polymerization contraction and stress [23].

BisGMA belongs to the group of aromatic dimethacrylate esters and is synthesized from epoxy resin and methyl methacrylate [24]. It is a relatively rigid molecule with terminal methacrylate groups that serve as sites for free radical polymerization and with two benzene rings present near the center. The high viscosity of BisGMA molecules is due to –OH groups and hydrogen bonds. However, its hardness and strength are the primary reasons for BisGMA being the most common organic molecule in the composite material composition. The rigid aromatic structure of BisGMA reduces the degree of polymerization contraction, while its low volatility and diffusion into oral tissues result in a higher modulus of elasticity and reduced toxicity [25]. The UDMA molecule in the form of a long linear chain contains one or more urethane groups and two methacrylate groups. While its molecular weight is comparable to that of BisGMA, it is characterized by greater flexibility and crosslinking capacity [26]. UDMA can incorporated into the organic matrix either alone or in combination with TEGDMA or BisGMA in order to reduce the overall viscosity of the resin material and increase the fracture resistance [27].

The fluid molecule 2-hydroxyethyl methacrylate (HEMA) has low molecular weight and has the capacity to sustain a reaction through unreacted double carbon-carbon C=C bonds even after most of the monomer crosslinking process has been completed [28]. As the long-chain TEGDMA molecule shows a relatively high degree of monomer-to-polymer conversion and thus leads to greater polymerization contraction, its percentage share in the material needs to be carefully balanced, as it also degrades some of its beneficial mechanical characteristics [25,29].

It is widely established that composite material networks based on TEGDMA are more heterogeneous compared to materials based on the BisGMA structure. The reason for this heterogeneity lies in the molecular composition and structure of the two monomers, TEGDMA and BisGMA. TEGDMA has a more flexible and smaller molecular structure, which leads to a higher degree of chain branching and irregularity in the material. On the other hand, BisGMA has a more rigid and bulkier molecular structure, resulting in a more ordered and uniform arrangement. In such heterogeneously structured molecules, the spaces between the micropores are larger, increasing the water storage capacity. The increased water sorption is a consequence of the greater flexibility of the network formed by the TEGDMA molecules relative to BisGMA as well as UDMA. Specifically, the hydroxyl groups of BisGMA form stronger hydrogen bonds with water molecules than do the urethane groups of UDMA or the ether groups of BisEMA molecules. Therefore, the percentage of BisGMA in the monomer has a significant effect on the mechanical performance of the material [25], with the available evidence suggesting that 70/30 is the most optimal BisGMA/TEGDMA volumetric ratio [30].

#### 2.1.2. Filler Particles

The incorporation of filler particles (quartz, barium silicate, strontium silicate, zinc silicate, lithium aluminum silicate, and yttrium and ytterbium trifluoride) significantly improves the performance of resin cement. The primary goal of these fillers is to reduce the percentage of the organic matrix and thus strengthen the material by improving compressive strength, tensile strength and modulus of elasticity [26]. Ferarri et al. reported that increasing the filler percentage from 10% up to 70 wt% reduced the bond strength between the luting material and the tooth structure while increasing material rigidity. Because this increase in rigidity led to polymerization stress, the authors recommend using resin cement with a filler content ranging from 10% to 30 w% [28]. According to reference data, the filler content varied between 31 vol% and 66 vol% [31] and between 17.36 to 53.56 vol% [32].

The reduction in the filler particle content inevitably leads to the weakening of the mechanical properties of the material, which in turn compromises its clinical performance. In the dental materials market, the focus is increasingly given to the formulation of luting agents that are also suitable for core build-up, allowing for simultaneous intraarticular post-cementation and core fabrication. However, the increased percentage of filler in these materials (which is necessary for core build-up restoration and retention) induces higher stress during polymerization, which is irreversible and ultimately results in lower bond-strength values when such materials are subjected to scientific research [28]. Depending on the size of the filler particles, resin-based materials can be classified into:traditional (1–50 µm), microfilled (0.04 µm), hybrid (0.1–20 µm), and nanofilled (1–100 nm) [29].

As voids between filler particles occur when the material contains particles of comparable sizes, particles of different dimensions should be included in its composition, and their overall share should be in the 30–70 vol%, i.e., 50–85 wt.%, range [26]. Moreover, the translucency of the restorative material should be similar to the translucency of natural teeth to improve the esthetics. The indicated statement relates to the resin-based restorative materials for which it is crucial to carefully match the color with the natural tooth. For glass ceramics materials, it is important to know that cement material can change the color of ceramic material. If this factor is not taken into account, the color of the cement underneath the veneer will modify its outward appearance and will compromise the esthetics of the applied restoration. In order to prevent such a mismatch, the dental material manufacturers offer the so-called try-in paste together with the resin-cement material [26].

#### 2.1.3. Silane

The establishment of a permanent bond between the filler particles and the organic resin matrix is made possible by the inclusion of a binding agent (silane) into resin cement [33]. Silane is a bifunctional molecule with the ability to establish a bond with the hydroxyl groups of inorganic filler particles as well as the methacrylic groups of the organic resin matrix. The most commonly used binding agent, gamma-methoxypropyltrimethoxy silane (gamma-MPS), belongs to the group of organosilanes [20]. Silanes can be as pre-activated solutions (single-phase) and two-component systems. Two-component silanes have to be mixed in order to initiate the hydrolysis reaction. Silane primers come in two parts, where one part contains the silane and the other part contains a catalyst or activator as this approach is often used to ensure the freshness and effectiveness of the silane treatment. Pre-activated silane primers exhibited a higher rate of hydrolysis than a two-component primer and the stability of pre-activated silane primers appears to be compromised due to the formation of oligomers [34].

#### 2.1.4. Other Components

Resin cements also include polymerization initiators (mono- and dimethacrylate monomers). The chemically polymerizing resins are sold in the form of two pastes, one of which contains benzoyl peroxide as an initiator and the other an aromatic tertiary amine as an activator. When the two pastes are mixed, amines react with benzoyl peroxide, forming free radicals and initiating an addition-type polymerization reaction [26]. The most commonly used tertiary amines are N,N-dimethyl-p-toluidine and N,N-dihydroxyethyl-p-toluidine, whereas N,N-dimethyl-p-toluidine is used less frequently due to the risk of discoloration [20]. On the other hand, light-cured materials are usually sold in the form of a single paste containing a photosensitive initiator and activator which do not react until the paste is exposed to light with a wavelength of about 468 nm. The resulting photosensor excitation enables interaction with the amine that leads to free radical formation [35]. Light-cured resins respond to this particular wavelength because they contain the diketone photoactivator camphorquinone (Norrish Type II photoinitiator) which is connected to the tertiary amine N,N-dimethyl aminoethyl methacrylate (DMAEMA) and exhibits the absorption maximum at 468 nm [36]. However, the contribution of camphorquinone and amine to the material composition should not exceed 0.2 wt.% and 0.15 wt.%, respectively [26]. Recent evidence also indicates that the inclusion of amine-free Norrish Type I photoinitiators, such as diphenyl (2,4,6-trimethylbenzoyl) phosphine oxide (TPO) or germanium-based Ivocerin, i.e., Bis-(4-methoxybenzoyl) diethylgermanium, improves color stability. Although both TPO and Ivocerin exhibit maximum absorption in the violet range, they remain sensitive in different parts of the spectrum. Thus, to polymerize restorative materials featuring a combination of these Type I and Type II initiators, lightpolymerizing units that operate at wavelengths in the 400–500 nm range should be used [37].

Moreover, to avoid the risk of spontaneous polymerization of methacrylate monomers, polymerization inhibitors such as monomethyl hydroquinone ether (MEHQ), butyl hydroxytoluene (BHT) and hydroquinone are also included in the composition of resin cement. These inhibitors show a strong reactive potential towards free radicals. As free radicals are formed when a small amount of material is exposed to light, because inhibitors react with free radicals faster than free radicals react with monomers, the polymerization chain does not commence until the entire amount of inhibitor has been consumed. The recommended BHT concentration is 0.01% and is sufficient to improve the material shelf life as well as extend the working time [26].

## 3. Polymerization Degree of Resin Cements

Free-radical polymerization is a feature of most polymers. In dentistry, resin-luting materials undergo a conversion of organic monomers into a crosslinked polymer structure [38]. Polymerization of resin cement depends on several factors. Among them, the type and percentage of filler particles, the type of organic resin matrix, the concentration and type of polymerization initiator, the intensity of emitted light and the exposure time are the most significant [21,39,40,41]. The exposure time is directly related to the monomer conversion degree, which is inversely proportional to the distance from the polymerization light source [7]. Moreover, as different initiator systems favor different types of polymerization reactions, and the presence of acid monomers in the composition of self-polymerizing cement reduces the polymerization efficiency, the inclusion of special activators or initiators such as sodium sulfate is advised [42]. As oxygen also has the capacity to slow down the polymerization reaction, it is important to be aware of its inhibitory effect [26].

In the literature, the degree of conversion (DC) is defined as the percentage of double carbon−carbon C=C bonds of the monomer that transform into single C−C bonds of the polymer and is calculated as the ratio of double C=C bonds in the polymerized and unpolymerized material [43] based on the following equation:

DC = [1 − R_polymerized_/R_unpolymerized_] × 100 (1)

where:

DC = polymerization efficiency or degree of monomer conversion (in %)
R = ratio of the peak area at 1638 cm^−1^ and 1608 cm^−1^ in the polymerized and unpolymerized material.

**Figure 3:**
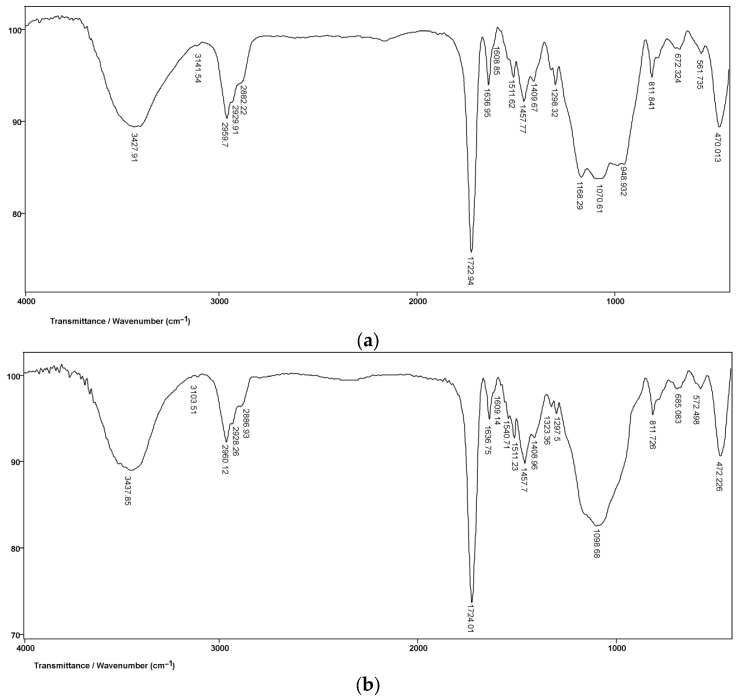
(**a**) FTIR spectrum produced by the Maxcem Elite resin before polymerization [22]. (**b**) FTIR spectrum produced by the Maxcem Elite resin after polymerization [22].

Aliphatic C=C bonds in the polymerized and unpolymerized material correspond to the peak located at 1638 cm^−1^ whereas the aromatic C=C bonds in the unpolymerized material give rise to the peak at 1608 cm^−1^. Given that the aromatic C=C bonds do not undergo changes during the polymerization reaction, the peak located at 1608 cm^−1^ is taken as an internal standard for the purpose of calculating the degree of monomer conversion (Figure 3a,b) [22,44].

Theoretically, during the polymerization process, all monomer molecules are converted into polymers. However, as in dimethacrylate monomers a certain percentage of unreacted double C=C bonds remains in the polymer, and the polymerization efficiency varies between 55% and 75% [26], even though 80.34% has been reported for self-polymerizing materials. While the polymerization efficiency of double-polymerizing cement is much lower at 73.58%, both are satisfactory values for these groups of materials [22].

In clinical settings, the chemical structure, the degree of monomer conversion and the kinetics of the polymerization reaction are the key determinants of the monomer mechanical performance and leaching potential [45,46,47]. Specifically, the degree of monomer conversion affects the mechanical characteristics and chemical stability of resin cement [21,43]. Thus, it is an important determinant of the physico-mechanical strength of the newly formed polymer [28,43,47,48], whereby a lower polymerization efficiency may result in altered biomechanical properties of the material. Extant evidence indicates that it reduces hardness as well as resistance to fracture and wear, while increasing hydrolytic degradation, and leading to a significant release of residual monomer, thereby reducing the level of material biocompatibility. Hence, when the level of conversion is far below the expected, a weaker bond between the material and the tooth structure is inevitable [49].

The degree of conversion of luting materials based on resins that have BisGMA in their composition is dependent on the amount of TEGDMA, whereby the greater proportion of TEGDMA improves the conversion degree due to the greater mobility and reactivity of its molecules [50,51,52]. The presence of TEGDMA is also beneficial because, after light activation, the polymerization process continues for the next 24 h, at the end of which the maximum degree of monomer conversion is achieved [53,54].

**Figure 4:**
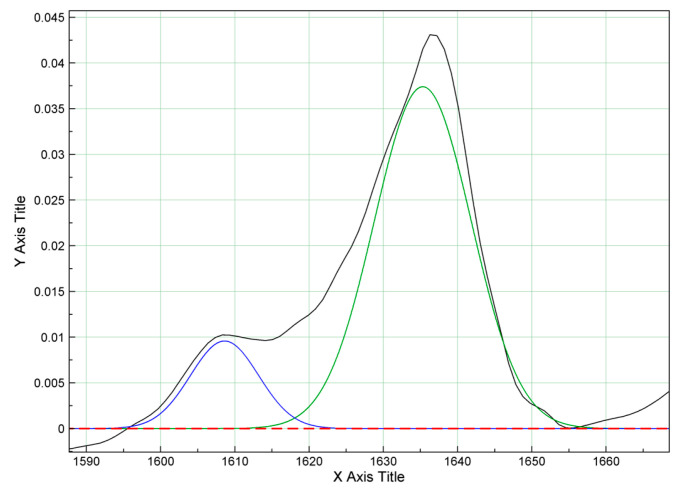
Fourier transform IC spectrum [22]. Different colored lines showed different infrared spectrum.

Unreacted monomer leaching has a potential biological significance, especially in the case of TEGDMA, which has been shown to cause changes at the DNA level of mammalian cells [55,56]. Extant evidence further indicates that unreacted monomers can stimulate bacterial growth in the immediate vicinity of the restoration and induce allergic reactions in some patients [25]. Thus, it is essential to determine the degree of polymerization efficiency which is usually executed by performing micro-Raman spectroscopy, Fourier transform infrared spectroscopy (Figure 4), differential thermal analysis (DTA), or differential scanning calorimetry (DSC) measurements [22,44,45,57,58,59].

Monomer crosslinking into solid polymer chains induces changes in the cement material, which results in volumetric contraction [25,60]. Under unconfined conditions, such volumetric contraction can be compensated by the flow of composite materials, while in constrained conditions, where the cement is in contact with the dental tissues, the volumetric contraction induces internal stresses in the material [60]. Volumetric contraction is determined from the proportion of filler particles and the type of organic resinous matrix [25]. The linear correlation between volumetric contraction and the amount of converted double C=C bonds was first observed by Loshaeck in 1953, who established that a mole of converted double C=C bonds into single C-C bonds was associated with a volumetric contraction of 23.0 cm^3^/mol [61]. These findings should be taken into account, as an ideal resin-based luting material should have minimal polymerization contraction and optimal monomer-to-polymer conversion [25,62].

## 4. Polymerization Mechanism of Resin-Based Dental Luting Materials

According to the polymerization reaction mechanism, resin cements can be classified into self-cure, light-cure and dual-cure categories (Table 2) [5,45,63].

Resin-based luting materials that polymerize chemically (self-cure) are two-component materials that contain a tertiary amine as an activator and benzoyl peroxide as an initiator. The incorporation of air bubbles during the manual mixing process leads to the formation of pores that weaken the material structure, as the trapped oxygen inhibits the polymerization reaction. Still, the main drawback of chemically-activated materials is the clinician’s inability to control the working time after mixing the constituent components [26].

These issues can be avoided by using materials that require photopolymerization, i.e., must be exposed to light of specific energy to initiate a photochemical reaction in the monomer. In these light-cured cements, adequate photopolymerization is critical in terms of optimal mechanical performance, biocompatibility and color stability [35]. Thus, tertiary amine is included in their composition and acts as a co-initiator that reacts with an activated photoinitiator to create free radicals. As the presence of camphorquinone results in undesirable yellow discoloration, its concentration is limited, even though this can reduce the polymerization efficiency [64].

These drawbacks can be overcome by the adoption of photoinitiator systems such as 1-phenyl1,2-propanedione (PPD) and octyloxy-phenyl-iodonium hexafluoroantimonate (OPPI), which have been shown to improve the polymerization kinetics while reducing the yellow effect in the polymerized material [35]. Light-cured cements are easy to handle and benefit from a controlled setting time, but their reliance on photopolymerization can be an issue when light penetration to all regions of the tooth structure (such as the intraarticular space) cannot be ensured [7]. An ample body of evidence also shows that the light beam weakens as it passes through the restoration material, making it difficult to achieve the intensity required for the polymerization of resin cement in deep and inaccessible parts of the tooth preparation. Consequently, a less successful and efficient polymerization of the resin cement is expected, which may lead to increased hydrolytic degradation, microleakage and the presence of secondary caries [5]. The aforementioned challenges can be mitigated by combining chemical and light-induced polymerization [13], which requires a dual polymerizing catalyst [65,66]. Namely, as a general rule, dual-cure cement polymerization commences with photoactivation, followed by chemical polymerization. Photopolymerization ensures the initial stability of the material, while chemical polymerization is responsible for achieving the most optimal material properties that can be sustained over time by enabling satisfactory polymerization, especially in regions that are not accessible to the applied light. Therefore, in clinical practice, the manufacturer’s instructions are strictly followed for each cement material [67]. The use of dual-polymerizing cement is thus indicated when insufficient light polymerization and inadequate monomer conversion are anticipated, such as during the cementation of intra-canal fiber posts [68]. Most dual cements have mix tips to improve the mix between the two parts and eliminate the hand mix, which is mostly responsible for inserting air bubbles [7].

Dual-cure resin cement can be conventional or self-adhesive [13]. As the application of an adhesive agent is necessary to achieve the desired strength of the bond between the cement and the tooth structure, this further complicates the already technically sensitive process of cement application. In practice, it is essential to follow proper isolation techniques to ensure optimal bonding between the tooth surface and applied cement. Proper isolation prevents any potential contamination that could compromise the bond strength. It is important to maintain a clean and dry field during all types of cementation procedures to achieve successful outcomes [69]. These challenges can be avoided by using self-adhesive cements, which involve fewer steps [70]. In addition to the simplification of the entire procedure and thus shorter work duration, self-adhesive resin-based cements benefit from adequate adhesion to dental tissues, especially dentin, without the need for a bonding agent, which is very important for the parts of the tooth where the application of the adhesive agent is difficult, such as the intra-radicular space when cementing intra-canal fiber posts [2,37,47].

Self-adhesive resin-based cements have multi-functional phosphoric acid methacrylates in their structure, which react with the hydroxyapatite in the teeth (Table 3) [65,66]. The polymerization reaction is a free-radical type and is initiated by a photoinitiator or redox system [8]. According to the information provided by the manufacturers, these cements contain acidic and hydrophilic monomers that simultaneously demineralize and infiltrate enamel and dentin, creating a strong bond. The organic matrix of some of the cement available on the market is based on novel multifunctional methacrylate phosphoric acid monomers, while others are based on carboxylic acid groups. An ample body of evidence shows that, in a specific concentration, acidic monomers successfully demineralize dentin and condition both dentin and enamel, thus enabling adhesion to dental tissues through a micromechanical process [47,71,72,73]. However, the concentration of acid monomers must be high enough to guarantee adequate demineralization and bonding with enamel and dentin, as well a sufficiently low to avoid additional hydrophilicity in the polymerized material [9].

Several authors have also cautioned that, owing to the potential incompatibility of self-polymerizing and dual-polymerizing cement and dental adhesives (in terms of the unfavorable reaction of residual acid monomers and aromatic tertiary amines), the entire polymerization process can be compromised. Accordingly, they advocate the use of adhesives with self-curing activators or chemical co-initiators such as sodium sulfate [39,74,75]. Indeed, sodium sulfate is already present in most of the available commercial adhesive preparations based on two-component “etch and prime” technology [75]. As the correct positioning of most currently used dental restorations depends on the appropriate adhesive interface preparation and management, dental practitioners must strictly adhere to the clinical protocol as well as possess adequate knowledge of the materials and substrates adopted for this purpose. Thus, continued training is mandatory to keep abreast of the latest developments in dental research and practice, including the specifics of various adhesive systems, and how to capitalize on their strengths while mitigating the impact of any weaknesses. In particular, they must be aware of the issues that may arise when using resin cement and adhesives produced by different manufacturers, which may not be compatible [76].

As restorations made of alloys or oxide ceramics do not allow the passage of light, the cement can only be chemically polymerized [77]. Hence, it is important to determine when the adhesive agent should be polymerized in relation to the application of cement material [39]. Given that there is presently no consensus regarding this procedure, and manufacturer instructions differ, in clinical practice, it is difficult to ascertain whether it is necessary to polymerize before applying the cement material, or leave the adhesive unpolymerized [75]. Still, it should be noted that a thicker polymerized layer of adhesive can make correct restoration positioning difficult, which would lead to problems in marginal closure and necessitate subsequent occlusal corrections [39]. The results reported by Coelho Santos et al. indicate that pre-curing the adhesive results in a thicker adhesive film, which in their study ranged from 5.7 to 14.8 microns. However, these authors also observed that, along the internal boundary of the indirect restoration, film thickness varied considerably. On the other hand, for adhesives that were not pre-cured prior to their use, no adhesive film could be distinguished from the resin-luting layer [78].

## 5. Polymerization Contraction of Resin-Based Dental Luting Materials

Polymerization contraction leads to the development of stress on the contact surface between the dental structure and luting material as well as on the restoration and the luting material, which can result in interfacial debonding, caries development and postoperative fragility [12]. Thus, extensive research has been conducted on the ways of mitigating this issue, including alterations to the resin-based filler by incorporating polymeric nanogels or epoxy oligomers, modifications to the resin matrix by using silorane resin and epoxy resin systems, and incorporation of unique low-shrinkage monomers in the material composition [79,80,81,82]. The most recent efforts in this context involve combining UDMA with triethylene glycol divinylbenzyl ether (TEG-DVBE) to obtain monomers that are less prone to polymerization contraction [82], as UDMA has high molecular weight monomer and high crosslinking potential, while TEG-DVBE is an ether-based diluent monomer characterized by lower viscosity [12]. While these achievements are noteworthy, it is expected that further attempts at minimizing the contraction during monomer polymerization will be made in the future.

The amount of stress in the material arising from the internal stress can be predicted by calculating the C factor, which Felizer defined as the ratio between bonded and unbonded cavity surfaces [83]. Accordingly, a high C factor indicates the presence of significant stress in the material, which undermines the bond strength between the material and dentin [60,84]. When cementing canal posts, inlays and crowns, cement is applied in a very thin layer characterized by a high C factor, which is sufficient to induce stress, and thus compromise the restoration retention, resulting in microleakage. According to Bouillaguet, these issues arise because the endodontic C factor is greater than 200, while the coronary C factor ranges from 1 to 5 [75,85]. As polymerization contraction depends on the C factor and the volume of composite material, polymerization contraction is particularly an issue for less viscous composite materials and can exceed 6% due to the lower proportion of filler particles (typically below 50 wt.%) [75].

Contraction stress and modulus of elasticity are directly proportional to the filler content [85]. Therefore, the TEGDMA/BisGMA ratio exerts a significant effect on both polymerization contraction and monomer conversion. Both chemically- and dual-polymerized cement exhibit lower contraction rates and stress values compared to light-polymerized cement due to the slower progression of the polymerization reaction. However, available evidence indicates that modifications of the polymerization regime through, for example, the application of “soft-start” polymerization and pulse-delay polymerization might reduce the occurrence and extent of contraction stress [86]. Soft-start polymerization is a two-phase polymerization technique that involves initial polymerization using light energy of lower intensity that gradually increases to the final full intensity [87,88]. On the other hand, pulse-delay polymerization involves a short initial exposure to light energy followed by a pause of at least 60 s before the final light exposure [89,90].

## 6. Characteristics of Resin-Based Dental Luting Materials

As resin cements are in a close and prolonged relationship with the pulpo-dentine complex, their biocompatibility is of great importance, especially when only a thin layer of dentin remains. Additionally, some of the structural components of resin-based cement can diffuse out of the material into saliva and be ingested, or can pass through the blood vessels of the pulp into the bloodstream [61]. In this context, it is also necessary to consider that the incomplete polymerization reaction of dimethacrylate monomers comprising composite cement leads to the leaching of unreacted monomers such as HEMA or TEGDMA, potentially exerting cytotoxic effects on the pulp and gingival cells. Extant evidence suggests that such undesirable effects may lead to hypersensitivity reactions in some patients [15] and even facilitate the development of cariogenic bacteria [91]. Extant research, however, shows that due to its easy solubility in patients’ saliva, TEGDMA has a greater cytotoxic potential compared to other dimethacrylate monomers [92,93,94]. We concur with the statements made by other authors who have evaluated these monomers in other cells, such as pulp cells and fibroblasts or macrophages. Specifically, as HEMA is a hydrophilic monomer, while TEGDMA is hydrophobic, its greater cytotoxicity may be ascribed to its higher cellular uptake and permeability. This assertion is consistent with a prior report indicating that monomer cytotoxicity arises due to the alterations in cellular permeability caused by changes in the cellular membrane lipid layers [95].

Moreover, the impact on odontoblast cells occurs already in the first week after cementing the restoration and it may persist for up to two months [96].

The main advantages of resin cements compared to conventional cements are bond strength to dental tissues and superior color, along with reduced solubility, improved mechanical properties and the possibility of photopolymerization or photo-chemical polymerization [97]. Thus, even though an ideal cement material is unattainable in practice, resin cement fulfill many of the required criteria, especially mechanical properties that will provide adequate resistance to masticatory forces and material stability in terms of pronounced resistance to degradation in the oral environment. Degradation of materials in the oral environment can be mechanical or chemical and is directly dependent on the chemical structure of the material and the prevailing conditions in the oral environment [97]. Therefore, the clinical performance of materials is established in terms of bond strength, solubility and hardness, which are usually determined under laboratory conditions [98]. To prolong the restoration longevity, the luting material should be impermeable to oral fluids and resistant to dissolution. In addition, it must ensure adequate restoration retention as well as tooth vitality, while preventing microleakage and secondary caries, all of which are also associated with cement water sorption [31]. Water sorption and solubility also impact the hardness and biocompatibility, as well as the dimensional and color stability of resin-based cements [72]. Therefore, in clinical practice, the aim is to achieve a functional connection between resin-based cement and dental tissue, which can be challenging and time-consuming, since the preparation of the dentin’s hydrophilic surface for the application of hydrophobic cement is a technically sensitive procedure that requires expertise. This process can be facilitated by using cements that are highly resistant to bending, while exhibiting a lower degree of polymerization contraction, as well as low water sorption and solubility [5,29].

From a clinical perspective, one of the primary requirements for resin cements is a suitable viscosity. This facilitates the correct positioning of the indirect restoration on the tooth without applying excessive pressure and also minimizes or eliminates the need for post-cementation adjustments or reocclusion [7].

## 7. Curent Perspectives

In the oral environment, indirect restorations cemented with resin cements are exposed to various masticatory forces and loads. Therefore, the layer of resin cement must withstand the shearing forces and prevent restoration displacement as well as preserve the connection between the restoration and the tooth structure [81].

One of the significant properties of composite resin cement is radiopacity. This property allows for the identification of areas where excess material may have leaked out and passed under the gingival margin. Therefore, composite resin cement should exhibit a radiopacity equal to or greater than that of dentin. It is worth noting that some of the most commonly used composite resin cement present a radiopacity higher than dentin, in accordance with ADA recommendations [6].

In modern dentistry, the aim is to simplify the cementing procedure involved in various restorations. Application of conventional resin cement involves a greater number of operative steps because it is necessary to prepare the dental structure. Each phase should be approached carefully while strictly adhering to all manufacturer’s instructions because any form of improvisation may jeopardize the clinical success of the restorative procedure. In this sense, the use of self-adhesive resin cement simplifies the entire work protocol [10,30,59,66]. While both groups of cements produce strong bonds with the tooth structure some results related to the conventional cements indicate that resin cements that are applied in combination with a bonding agent do not achieve as strong a connection with the tooth structure [45,65,66]. Contrary to these findings, available evidence indicates that etch and rinse adhesive systems lead to superior bond strength relative to self-etching bonding agents [99]. This is particularly important when restorations are placed on implants, as cement penetration into the peri-implant tissue can result in implant failure. As durable sealing between abutment preparations and the metal framework requires the use of water-soluble cement exhibiting lower disengagement resistance when the available space for cement is limited, it is necessary to carefully evaluate the indications for the application of non-water-soluble resin materials when cementing retained implant restorations [90].

Posts and cores are commonly used in endodontically restored teeth. Currently, glass fiber is the most frequently used post system due to its superior adhesive bonding and tooth fracture strength, as well as improved translucency and thus esthetics. Given that zirconium posts are also available on the dental market, it is essential to determine the most optimal cement material for cementing those posts. The current recommendation is to use resin cement rather than conventional cement [99].

When considering the biological acceptability of different composite resin cements, the primary concern is preventing the leaching of unreacted monomers and its potentially harmful effects on human health. The findings of a significant body of research in this domain indicate that the use of resin cement with greater polymerization efficiency reduces the risk of leaching. Substances that are released from resin materials have been shown to exhibit cytotoxicity, which is particularly pronounced on human oral fibroblasts derived from gingiva, dental pulp, and periodontal ligament. However, this impact is the greatest for BisGMA, followed by UDMA, TEGDMA, and finally HEMA. Moreover, findings yielded by extant implantology research indicate that, as substances eluted from resin cement have the potential to disrupt osteoblastic homeostasis, these unfavorable conditions could give rise to peri-implant bone destruction. Accordingly, their correct use in clinical practice is mandatory, including the complete removal of all cement residues and ensuring that sufficient polymerization has been achieved [91,100].

However, the influence of polymerization modulus on leaching requires further investigation. Available evidence indicates that, whenever possible, light curing should be utilized for dual-cure resin cement as it prolongs indirect restoration stability and reduces cellular cytotoxicity [97]. In particular, light-cured resin cements are recommended for aesthetic (highly translucent) restorations of <1.5 mm thickness as this results in enhanced shade matching as well as longer-lasting color stability compared to dual- and self-cured alternatives. While the latter varieties are necessary for thicker aesthetic restorations (2–3 mm), light-cured cement has been found to yield greater early hardness than the dual-cured type in restorations involving feldspathic and polymer-infiltrated ceramics irrespective of the substrate thickness [101].

## 8. Future Perspectives

Future research endeavors are expected to target further improvements in the performance of resin cement, especially the bond between ceramic restorations and resin cement, which requires adequate preparation of the inner surface of the ceramic restoration. Ceramic restorations containing a glass phase must be acid etched prior to bonding to the tooth structure to optimize the surface texture for resin cement adhesion. In extant research, the highest bond strength between an adhesive-resin cement and glass ceramics was achieved by treating the porcelain’s surface with 5–9.5% hydrofluoric acid, while using 37% phosphoric acid and silane as the etching and coupling agent, respectively. However, the same authors found that the acid treatment outcome depends on the type of ceramic material, the conditioner concentration and the etching duration [102]. Glass ceramic bonding can be effectively achieved through hydrofluoric acid etching and the application of a ceramic primer containing a silane coupling agent because glass ceramics incorporate the silica base. Accordingly, as the hydrofluoric acid dissolves the glass matrix, micromechanical retention is achieved by the remaining porous structure. The inner surface of restorations containing silicate ceramics (feldspat and glass ceramics) is prepared by partially dissolving the glassy matrix with hydrofluoric acid. For this purpose, the use of hydrofluoric acid at different concentrations (ranging from 4% to 9.8%) is recommended, whereby the duration of its application depends on the presence of the crystalline phase. Ceramics with a higher crystal content require a shorter etching time and a lower acid concentration [103]. Ceramic restorations made of glass-infiltrated ceramics are prepared by sandblasting with Al_2_O_3_ particles, with an average size of 50 µm, under 380 kPa pressure for 10–15 s. In both cases, surface preparation is followed by silane application to connect the organic component of the cement with the inorganic component of the ceramic [104].

Thus, to promote their greater adoption, it is necessary to establish a simplified and more efficient method for their inclusion in the adhesive application protocol, aiming to reduce clinical operation time without compromising bond strength and durability. Several authors are of the view that treatment with additional silane prior to the application of universal adhesives should be considered, as it has been shown beneficial for the effectiveness and longevity of ceramic–resin bonding [105].

Although in clinical practice surface treatment for glass-ceramic restorations typically involves hydrofluoric acid etching followed by glass-ceramic primer treatment, this adhesion strategy requires at least three work steps, making it not only time-consuming but also highly dependent on the clinician’s capacity to perform all steps with a high degree of precision. To mitigate these issues, manufacturers have invested considerable effort into luting procedure simplification, and have developed universal adhesive systems for bonding to glass-ceramics [106].

At present, there is no consensus among researchers and clinical practitioners on the application of an adhesive layer on glass-ceramics after etching with hydrofluoric acid and the use of a silane-coupling agent [107]. However, based on the investigation of the feasibility of employing an Er:YAG laser to pretreat glass-ceramic surface and the effect of this strategy on the bonding strength and marginal adaptation between the restoration material and dentin, the usage of laser energy for this purpose is beneficial [108].

For polycrystalline ceramics, authors of more recent studies advocate the adoption of primers based on 10-methacryloyloxydecyl dihydrogen phosphate (10-MPD) as their use improves the adhesion between zirconia and composite resins, especially when preceded by alumina sandblasting [109]. Surface pretreatment is required as a durable bond with zirconia can only be achieved after airborne-particle abrasion using alumina (Al_2_O_3_) particles followed by the application of an agent containing special (phosphate) monomers, MDP in particular. MDP is a subject of extensive research and is a constituent of a variety of adhesives, including most of the universal adhesives [76,110]. Universal adhesives are particularly popular in clinical practice as they are manufactured as a single-bottle, no-mix, adhesive system that can be used in total-etch, self-etch, or selective-etch mode for direct and indirect restorations. As they are compatible with self-cure, light-cure, and dual-cure resin-based cements, universal adhesives can be used not only to bond to dentin and enamel but as adhesive primers on substrates such as zirconia [111]. However, when developing a universal adhesive, it is important to balance their hydrophilic and hydrophobic characteristics, as the monomers need to initially be sufficiently hydrophilic to wet, infiltrate, and interact with the dentin, but their hydrophilicity should decline once they are polymerized to prevent water sorption, as this could lead to hydrolysis and breakdown of the adhesive interface over time [111].

Available data also suggests that sandblasting is a preferred choice for restorations based on hybrid ceramics, while hydrofluoric acid is recommended for glass ceramics and those incorporating resin nanoceramics reinforced with nanoparticles [112].

In clinical practice, dual-cured resin cements are typically used to lute indirect restorations because they exhibit the advantages of light activation (thus allowing for clinician-controlled restoration stabilization) as well as those of chemical activation (thereby ensuring the required degree of polymerization in regions that are inaccessible to the applied light). However, as high cement conversion in those regions necessitates an increased concentration of redox initiators, working time is markedly reduced while the potential for discoloration is increased, thereby compromising the luting outcome. These issues were investigated by Faria et al., who demonstrated that experimental dual-cured cement containing 20 wt.% of thiourethane extended the working time, improved conversion and reduced polymerization stress, without compromising the mechanical properties of the restoration material [109]. Although these findings are encouraging, dual-cure cements present the risk of oxidation-induced discoloration due to their high amine-based co-initiator content, which may compromise the esthetic outcome. To overcome these notable drawbacks, researchers and manufacturers have proposed alternative formulations, including amine-free resin cement, but further research is required to establish if these changes lead to greater color stability [112].

Some authors are also of the view that low-shrinkage-stress resin-based cement with antibacterial properties could be used at the tooth-restoration interface to reduce the microleakage risk and avoid recurrent caries, thereby enhancing the longevity of fixed dental restorations. However, as such cements are not presently available in the market, a novel low-shrinkage-stress resin-based cement containing dimethylaminohexadecyl methacrylate (DMAHDM) was recently developed. The findings yielded by investigating its mechanical and antibacterial properties confirmed that, owing to the positively charged quaternary amine, N+, this type of quaternary ammonium methacrylate (QAM) has the capacity to interact with the negatively charged bacterial cell membrane, which causes an electrical imbalance that is conducive to bacterial cell disruption and cytoplasmic leakage. In extant studies, other resin-based materials based on DMAHDM (such as composites, sealers, adhesives, and cements) were developed and tested and were demonstrated to exhibit strong and long-lasting antibacterial effects against cariogenic biofilm [12]. Thus, when clinicians assess the risks and benefits of dental materials, they should also consider biocompatibility [113].

In this context, it is worth noting that some recently developed resin cements are capable of imparting “self-healing” properties of the organic matrix, rendering it more resistant to cracks and fractures. This beneficial effect is achieved by embedding microcapsules containing healing liquid into the composite material. Accordingly, polymer cracking will cause the microcapsules to rupture and release the healing liquid into the crack planes, where it is exposed to the catalysts in the polymer matrix, which ultimately results in polymerization, effectively filling the crack. According to Hong Leung who developed resin composites using triethylene glycol dimethacrylate-N and N-dihydroxyethyl-p-toluidine healing liquid encapsulated in poly(urea-formaldehyde) shells, this material was successful in mitigating newly-initiated fractures [24].

The most recently developed group of dental materials are denoted as “bioactive” because they can activate dental tissue repair mechanisms, as well as elicit a positive response from the dental tissue [114]. In this context, Activa™ BioActive-Restorative (Pulpdent Corp., Watertown, MA, USA) is particularly noteworthy as this bioactive restorative material exhibits the strength and esthetics of composites while possessing the advantages of glass ionomers. The purpose of the bioactive ionic resin matrix is a high release and recharge rate of calcium (Ca^2+^), phosphate (PO_4_^3−^) and fluoride (F^−^) ions, while the rubberized resin provides the required strength and durability. Moreover, it contains reactive glass ionomer fillers that, in addition to releasing large quantities of fluoride, replicate the physical and chemical attributes of natural teeth [115]. At present, however, the use of bioactive materials is limited to pediatric dentistry, but the need for a cement material with similar characteristics is unquestionable. Thus, it is likely that greater research efforts will be dedicated to the development of bioactive dental cement materials in the future.

## 9. Conclusions

The current dental material market offers a wide range of resin cement with diverse and continually advancing properties. While this is certainly beneficial, such an extensive choice of materials can be confusing even for dentists with many years of experience. Thus, it is extremely important to provide reliable and succinct guidelines on the physical, mechanical, biological and aesthetic properties of the most commonly used materials to allow dental practitioners to make informed decisions regarding specific indications.

## Figures and Tables

**Table 1:** Characteristics of dental luting materials (compiled from the findings reported in articles cited under reference numbers [3,13]).

Dental Luting Materials	Compressive Strength (MPa)	Flexural Strength (MPa)	Modulus of Elasticity (GPa)	Water Solubility (wt%)
Zinc-phosphate cement	104	5.5	13.5	0.06
Polycarboxylate cement	55	6.2	5.1	0.06
Glass-ionomer cement	86	6.2	7.3	1.25
Composite resin-based cement	70–172	42	2.1–3.1	0–0.01

**Table 2:** Some of the most commonly used dental resin-based luting materials in everyday dental practice (based on the data related to the studied cement materials sourced from the Safety Data Sheets/Free SDS Database).

Resin-Based Luting Material	Manufacturer	Polymerization Mechanism	Composition
**VARIOLINK II**	IvoclarVivadent AG, Schaan, Liechenstein	Dual-polymerizing cement Requires Excite DSC utilization	BisGMA, UDMA, TEGDMA
			Barium glass
			Ytterbiumtrifluoride
			Ba-Al fluorosilicate glass
			Dibenzoyl peroxide
**MAXCEM ELITE**	Kerr Scafati, Italia	Chemically polymerizing cement “Self-adhesive”cement	HEMA
			4 Methoxyphenol
			Cumene HydroPerOxide
			Titanium Dioxide
			Mineral fillers
			Ytterbium fluoride
**SPEEDCEM**	IvoclarVivadent AG, Schaan, Liechenstein	Chemically polymerizing cement “Self-adhesive”cement	UDMA, TEGDMA
			Barium glass
			Ytterbiumtrifluoride
			Dibenzoyl peroxide
**RELYX ARC**	3M ESPE, Landsberg am Lech, Germany	Dual-polymerizing cement Requires Single Bond Adper utilization	TEGDMA
			BISGMA
			SILANE TREATED SILICA
			REACTED POLYCAPROLACTONE POLYMER 2-BENZOTRIAZOLYL-4-METHYLPHENOL BENZOYL PEROXIDE
**RELYX VENEER**	3M ESPE, Landsberg am Lech, Germany	Light-polymerizing cement Requires Single Bond Adper utilization	BISGMA
			TEGDMA
			Titanium Dioxide
			Diphenyliodonium
			Hexafluorophosphate N,N-DIMETHYLBENZOCAINE

**Table 3:** Self-adhesive resin-based luting material and their chemical structure (based on the data related to the studied cement materials sourced from the Safety Data Sheets/Free SDS Database).

**RelyX U100** **3M ESPE**	Base: glass fiber, multifunctional methacrylate phosphoric acid monomers, dimethacrylates, silanated silica, sodium persulfate.
	Catalyst: glass fiber, dimethacrylates, silanated silica, p-toluene sodium sulfate, calcium hydroxide
**Maxcem Elite** **Kerr Corporation**	Resin matrix: GPDM, co-monomers (mono-, di-, and tri-functional methacrylate monomers), proprietary self-curing redox activator, photoinitiator CQ, stabilizer
	Filler load 67%wt: fluoroaluminiosilicate glass, fumed silica, barium glass, ytterbium fluoride

## Data Availability

Not applicable.
